# Who chooses alternative sources of information about childhood vaccinations? A cross-sectional study

**DOI:** 10.3389/fpubh.2023.1225761

**Published:** 2023-09-13

**Authors:** Rosa Katia Bellomo, Vito Cerabona, Azzurra Massimi, Giuseppe Migliara, Michele Sparano, Francesco Novello, Tiziana Schilirò, Roberta Siliquini, Paolo Villari, Corrado De Vito

**Affiliations:** ^1^Department of Public Health and Infectious Diseases, Sapienza University of Rome, Rome, Italy; ^2^A.S.L. City of Turin, Turin, Italy; ^3^Department of Public Health and Pediatrics, University of Turin, Turin, Italy; ^4^A.O.U. City of Health and Science of Turin, Turin, Italy

**Keywords:** vaccine hesitancy, parents, sources of information, health literacy, infodemic

## Abstract

**Introduction:**

Vaccine hesitancy can lead to problematic outcomes in terms of public health. A factor playing a fundamental role in this dynamic is the source of information considered by parents in the decision-making progress that leads to the acceptance or refusal of childhood vaccinations. This study aims to investigate the sources of information considered by the parents of children attending primary and secondary schools in two large Italian cities and to identify predictors that led to choosing alternative sources of information.

**Methods:**

An online questionnaire was administered to the parents of students attending elementary, middle, and high schools in Rome and Turin. Two validated tools were used: the Parent Attitudes about Childhood Vaccines Survey and the Vaccine Health Literacy of adults in Italian. Sources of information about vaccinations, trust toward the healthcare system, hesitancy and attitudes about COVID-19 vaccinations, were also investigated. A multivariable logistic regression model was built to identify predictors of the preferred sources of information on the topic.

**Results:**

Totally, 2,301 answers to the survey were collected from June to October 2021. Of these, 1,127 came from parents in Rome (49%) and 1,174 from parents based in Turin (51%) with a mean age of 47.7 years (±6.4). The majority of the respondents were mothers (81%), married (73%), with two or more children (70.5%). The multivariable logistic regression model results showed that fathers were more inclined than mothers to use alternative sources of information (OR 1.48, 95% CI 1.29–2.00). Moreover, a higher level of vaccine hesitancy was a strong predictor for choosing alternative sources of information (OR 2.45, 95% CI 1.73–3.46). The HLVa-it scores show that parents with a lower Vaccine Literacy (VL) were more inclined to use alternative sources of information.

**Discussion:**

Addressing health literacy issues and changing the official forms of communication could help improving vaccine acceptance. This study shows the importance of rebuilding a trusting relationship between patients and health care providers, which is fundamental in the fight against vaccine hesitancy.

## Introduction

Population attitudes toward vaccination are still highly controversial, with large pockets of resistance persisting in all countries (1). Apart from anti-vaccination activism, this phenomenon is largely part of a broader issue called vaccine hesitancy, identified – well before the pandemic – as one of the WHO’s top ten global health threats, and has become a major concern about hesitancy toward childhood vaccination campaigns (2). Vaccine hesitancy is complex and context-specific, varies by time, place, and vaccine, and is influenced by factors such as complacency, convenience, and trust (2). The COVID-19 pandemic, and the unprecedented vaccination campaign that followed, reinforced misconceptions about vaccines and caused a massive shift in the field of communication, with a significant increase in the use of alternative sources of information that seemed more reliable and responsive to many (3–5).

A significant factor to account for in this matter is the sources from which parents get information about vaccinations (6), particularly in the era of widespread mass communication and social media (7). Previous research on the topic showed how the extent to which parents searched for information about vaccination and how they received and assessed this knowledge were associated with their trust in the chosen source and also that parents generally considered mass media, for example newspapers, magazines, television and the Internet, as an essential source of information on the topic (8–10). When health literacy and opinions among parents vaccinating their children were investigated after the spreading of the fake news wave about MMR vaccination, it appeared that only 25% had spoken to their GPs about MMR, with a general feeling of either ‘abandonment’ or distrust toward the institutional sources of information, with parents often describing their relationships with the GPs as unhelpful and passive (11). In some cases, this behavior has led to a higher level of inadequate knowledge such as the one that was found among young adults who received medical information from family/friends about HPV vaccination (12). These findings, on one side, imply that the already existing official information is sometimes not adequate both in contents and language to answer the population’s doubts on the topic; on the other side, they show an increasing lack of trust toward governments and institutionalized actions of public health, with people preferring a ‘hive’ type of communication and narrative better than an old model of hierarchically generated expert metanarrative (13). Henceforth, in modern medical science, it is becoming more and more necessary to address the increasing complexity of relationships between patients and healthcare providers to prevent the spread of these tendencies, including vaccine hesitancy, which can have catastrophic consequences on public health (14). Previous studies underlined how sources of information on healthcare topics are deeply connected to this issue and can significantly influence the decision to comply to vaccinations, both regarding childhood and adulthood vaccination campaigns (15, 16).

The general population has always considered the Internet as a free source of information, so dubious parents often choose this instrument seeking answers to their questions, especially when they do not see their own or their children’s health provider as a reliable source of information. Compared to parents who did not use the Internet for vaccine information, those who used it were more likely to have lower perceptions of vaccine safety, vaccine effectiveness, and disease susceptibility and were more likely to have a child with a nonmedical exemption (7). Moreover, the rise of social media access and the total lack of control over the information shared on these platforms have favored the diffusion of many fake news and conspiracy theories, very often related to healthcare services and public health (17). Furthermore, on the internet, side by side with spontaneous aggregation groups of patients sharing the same doubts toward conventional medicine, there are a lot of alternative sources funded by people or companies with a solid background in social and communication sciences that are currently capitalizing the population’s fears and worries, playing a significant role in diffusion of misinformation, as it happened with the production of a documentary series advertised as a groundbreaking truth revelation within the largest anti-vaccination closed social network group (18). However, the relationship between misinformation and vaccine hesitancy is very complex. Some authors, after analyzing the association between vaccine hesitancy toward COVID-19 vaccine and misinformation, have developed a theory that the association between misinformation and hesitancy is not strictly a causal relationship (19). A situation could arise where people often adopt beliefs in conspiracy theories to justify preexisting views in the face of evidence that does not support them or use these theories to rationalize personal or political losses.

Understanding the decisional process and potential determinants underlying the parental behavior in this delicate matter is critical for planning effective health communication campaigns, increasing the childhood vaccination uptake and hence implementing population immunization. The aims of the present study are: (i) to investigate the sources of information taken into account by the parents of children attending primary and secondary schools in Rome and Turin to decide whether to vaccinate their children or not and (ii) to identify predictors that led to choosing alternative sources of information (tv, Internet, magazines) instead of institutional ones as their main base for decision making.

## Materials and methods

### Setting and participants

This cross-sectional study was conducted from June to October 2021. An anonymous survey was administered through Google Forms to parents of children attending elementary, middle, and high schools in Rome and Turin and kindergarten in Turin. Participating schools were sampled from the total of schools in Turin and from the total of schools in six different municipalities in Rome. Specifically, a sampling with probabilities proportional to the size of the school stratified by grade (Kindergarten, Elementary school, Middle school, High school) and by municipalities (for Rome) or districts (for Turin, eight districts) was applied to identify one school for each grade and municipality or district. The chosen municipalities represent both central and peripheral parts of Rome.

The participant sample size was estimated in 540, assuming a total population of 145.000 subjects in Rome, a proportion of the expected outcome of 30%, a margin of error of ±5%, a confidence interval of 95%, and a response rate of 60%.

The list of participants was retrieved using the electronic register of the schools they belonged to. The study protocol was approved by the Ethics Committee of Regione Lazio (reference number: 660/CE Lazio1).

Before the study, all parents were provided with information about the study’s methods, objectives and anonymity guarantees. A direct link to an online questionnaire was published on the electronic register personal page after obtaining consent to process sensitive data for the study. To minimize the risk of confidentiality breaches for participants, questionnaires were completed anonymously. Completed questionnaires were accessible only to the study investigators.

### Questionnaire

The questionnaire adopted was divided into four sections, two of which are composed of tools validated by the scientific literature: the Parent Attitudes about Childhood Vaccines Survey (PACVs) validated in Italian (20, 21) and the Vaccine Health Literacy degli adulti in Italiano (HLVa-IT, Vaccine Health Literacy of adults in Italian) (22).

The first section included a demographic assessment investigating the social characteristics of the sample (city, sex, age, number of children, children’s age, education level of both parents, occupation of both parents, nationality, religion, political tendencies and trust toward the national healthcare system). Also, parents were asked to rate the national healthcare system on a scale from 0 (meaning bad perceived quality) to 10 (representing excellent quality). Scores from 0 to 3 were defined as low quality, 4–7 as medium quality and 8–10 as high quality.

The second section included the PACVs, which is divided into three subsections investigating behaviors, safety and efficacy, and general attitudes toward childhood vaccinations. The raw score from the PACVs questions ranges from 0 to 30 and is converted to a 0 to 100 score using a simple linear transformation (20). A score ≥ 50 indicates a stronger tendency toward vaccine hesitancy (20). Two additional questions investigating the sources of information accessed about vaccines and the perceived need for further information on the topic were added to this section. The sources of information included were: TV, newspapers, Internet, healthcare providers (family doctors or pediatrician and medical doctors in general), vaccination centers, schools, family or friends, none, other (specified by the responder). For data analysis, healthcare providers (medical doctors, vaccinal centers), institutional websites (governmental and intergovernmental organizations, medical and healthcare organizations, hospitals, and academic medical institutions were considered as reliable sources of health information), scientific literature and schools were considered institutional reliable sources of information (IS), while friends/family, tv/newspapers, Internet and “none” were considered non-institutional sources (NIS).

The third section investigated the vaccine health literacy of adults through the HLVa-IT questionnaire, composed of three scales assessing the functional vaccine literacy (VL), the interactive or communicative VL and the critical VL, respectively. From a psychometric point of view, the questions of the functional VL scale are primarily about language capabilities involving the semantic system. In contrast, the interactive and critical VL scales evaluate cognitive efforts, such as problem-solving and decision-making. Each scale consists of 4 questions, providing a 4-point Likert scale response (4—never, 3—rarely, 2—sometimes, 1—often, for the functional questions; and 1—never, 2—rarely, 3—sometimes, 4—often, for the interactive and critical questions). Moreover, the HLVa-IT tool presents two filter questions before each section with a yes/no answer that allows the responder to proceed with the rest of the questionnaire in case of a positive response, identifying people who had the chance to read material about vaccination. Specifically, the first question assessed (before the functional scale items) whether the parent had ever read any informative material about vaccinations, while the second question, which filters the access to the communication and critical scales, assessed whether they had ever been advised to get a vaccination shot. The final VL score was calculated as the average of rates of each question (ranging from 1 to 4), with higher values corresponding to higher VL levels (scores <2 indicating a lower VL and scores ≥2 indicating a higher VL).

The fourth section concerned vaccine hesitancy for COVID-19 (10 items investigating inclination toward COVID-19 vaccination). Specifically, information about immunization coverage among parents and children (stratified in two age groups, older than 12 years and younger than 12 years) was collected, the reasons that led the parents to accept the vaccinations for either themselves or their children were investigated and the attitude toward future anti-COVID-19 shots administrations were assessed. In case of vaccine refusal for either the parents or the children, the reasons behind anti-COVID-19 vaccine hesitancy were evaluated. The last survey question investigated how the anti-COVID-19 vaccination campaign influenced trust or hesitancy toward vaccinations in general. The full version of the questionnaire is available in the supplementary material.

### Statistical analysis

Data were collected anonymously, entered into a database and processed with the statistical software STATA 17.0 (StataCorp, Lakeway Drive, TX). The statistical analysis aimed at the descriptive analysis of the population under study concerning socio-demographic and work variables and the responses to the proposed questionnaires. For categorical variables, absolute and relative frequencies were calculated, while means and standard deviations (SD) or medians with interquartile ranges (IQR) were used for continuous variables. The univariable analysis was performed through the Pearson’s χ^2^, or the Fisher’s exact test if appropriate. The Student’s *t* test and the ANOVA, or the Mann–Whitney and Kruskal-Wallis tests if appropriate, were used for continuous variables. Normality of continuous variables was checked through the Shapiro–Wilk test. A multivariable logistic regression model was built to identify predictors of alternative sources of information choice.

The covariates to be included in the model were chosen using a pV < 0.25 at the univariate analysis as a cut-off or based on their epidemiological relevance. The odds ratios (OR) and their 95% confidence interval (95%CI) were calculated for each independent variable. A value of *p* ≤0.05 were considered statistically significant, and all tests were 2-sided. The Hosmer-Lemeshow test was used to assess the goodness of fit of the logistic regression models (23). City of origin (0 = Rome, 1 = Turin), parental role (0 = mother, 1 = father), age (years, continuous), marital status (0 = single parent, 1 = married/cohabitant), profession (0 = employed, 1 = unemployed/housework), nationality (0 = Italian, 1 = other), religion (0 = believer, 1 = non-believers, 2 = would rather not answer/other), child’s school grade (1 = kindergarten, 2 = primary school, 3 = middle school, 4 = high school), vaccine hesitancy (0 = no, 1 = yes), educational level (0 = compulsory schooling, 1 = degree/post degree education), perceived healthcare system quality (0 = low quality, 1 = medium quality, 2 = high quality), self-perceived need for further information (0 = no, 1 = yes), HLVa-IT functional scale score (0 = having read material about vaccinations, 1 = score < 2, 2 = score ≥ 2) and HLVa-IT critical scale score (0 = having been advised to receive a vaccination shot, 1 = score < 2, 2 = score ≥ 2) were the covariates included in the regression model.

## Results

### Socio-demographic characteristics of IS and NIS users

Out of 22,760 invited participants to the survey, 2,301 parents completed the questionnaire, with a response rate of 10.1%, equally distributed between Rome (49.0%) and Turin (51.0%). The vast majority of our responders was Italian (96.3%) and only 3.7% were foreigners. Most responders had their children attending high school (44.4%), followed by elementary school (24.2%), middle school (22.4%), and kindergarten (9.0%, from Turin only). Moreover, they were more often female (81%), married or cohabitant (73%), and with two or more children (70.5%). The mean age was 47.7 ± 6.4 years. The general characteristics of the study population are reported in Table 1.

**Table 1:** Socio-demographic characteristics among parents using institutional (*N* = 1,663) and non-institutional (*N* = 638) sources of information.

	Institutional sources *n* = 1,663 *n* (%)	Non-institutional sources *N* = 638 *n* (%)	*p*-Value
City			
Rome	841 (74.6)	286 (25.4)	0.014
Turin	822 (70.0)	352 (30.0)	
Gender			
Female	1,380 (73.9)	488 (26.1)	<0.001
Male	283 (65.4)	150 (34.6)	
Age, years (*n* = 2,300)			
mean (SD)	47.5 (6.2)	48.1 (6.8)	0.056
Marital status			
Single	218 (66.9)	108 (33.1)	0.019
Married/Cohabitant	1,445 (73.2)	530 (26.8)	
Educational level			
Compulsory schooling	672 (71.5)	268 (28.5)	0.485
Graduate/Post-graduate	991 (72.8)	370 (27.2)	
Profession			
Employees	1,506 (72.65)	567 (27.35)	0.225
Unemployed/retired/housework	157 (68.9)	71 (31.1)	
Partner’s profession (*n* = 2,144)			
Employees	1,491 (73.5)	538 (25.5)	0.006
Unemployed/retired/housework	172 (63.2)	100 (36.8)	
Religion (*n* = 2,300)			
Believers	1,286 (73.1)	474 (26.9)	0.067
Non-believers	200 (66.7)	100 (33.3)	
Would rather not answer/other	176 (73.3)	64 (26.7)	
Number of children			
1 child	479 (70.5)	200 (29.5)	0.231
2 or more	1,184 (73)	438 (27)	
Child’s age			
0–5	65 (69.15)	29 (30.85)	0.122
6–11	534 (75.5)	173 (24.5)	
12–18	1,012 (71.1)	412 (28.9)	
>18	52 (68.4)	24 (31.6)	
School grade			
Kindergarten	219 (70.9)	90 (29.1)	0.010
Elementary school	368 (78.1)	103 (21.9)	
Middle school	820 (71.4)	328 (28.6)	
High school	256 (68.6)	117 (31.4)	
Political tendency			
Left	646 (72.3)	248 (27.7)	0.932
Moderate	296 (71.15)	120 (28.85)	
Right	252 (73.3)	92 (26.7)	
Prefers not to answer	469 (72.5)	178 (27.5)	
Perceived healthcare system quality			
Low	52 (52)	48 (48)	<0.001
Medium	1,004 (71)	410 (29)	
High	607 (77.1)	180 (22.9)	
Self-perceived need for further information			
No	1,014 (76.2)	316 (23.8)	<0.001
Yes	649 (66.8)	322 (33.2)	

Overall, 27.7% of parents reported to gather vaccine information from NIS rather than IS. Specifically, NIS users sought information mostly on tv and newspapers (39.5%), internet (34.3%) and friends and family (10.2%), while IS users consulted principally pediatricians and vaccinal centers (55.7%), and family doctors (38.0%). NIS users were more common among responders from Turin (30.0% vs. 25.4%), male (34.6% vs. 26.1%), single (33.1% vs. 26.8%), and with non-working partners (38.3% vs. 27.4%). Moreover, parents of children in high schools seemed to be more prone to refer to NIS than those of children attending other grades (31.4% vs. 29.1% for kindergarten, 28.6% for middle schools, and 21.9 for elementary schools). Regarding the perceived quality of the healthcare system, a low perception was associated with an increased use of NIS (48.0% vs. 29.0% for medium perception, and 22.9% for high perception). Similarly, a self-perceived need for further information on vaccines seemed to push toward NIS (33.2% vs. 23.8%). No differences in use of NIS were found for age, educational levels, profession, religious belief, political tendency, number of children, and age of child frequenting school.

### Attitudes, behaviors, and literacy toward vaccines among IS and NIS users

The attitudes, behaviors and literacy toward vaccines of the study population are reported in Table 2. Overall, the assessment of vaccine hesitancy through the PACV resulted in 93.1% of parents with a non-hesitant behavior. With regard to the choice of source of information, NIS were far more common among hesitant parents (52.2%) than non-hesitant (25.9%).

**Table 2:** Vaccinal intentions according to PACVs, HLVA-it scale, and Covid questionnaire.

	Official sources *n* = 1,663 *n* (%)	Alternative sources *n* = 638 *n* (%)	*p*-Value
PACVs score			
<50	1,587 (74.1)	555 (25.9)	<0.001
≥50	76 (47.8)	83 (52.2)	
HLVA-IT functional scale score			
Responded “no” to 1^st^ filter question	271 (65.3)	144 (34.7)	<0.001
1–2	67 (58.8)	47 (41.2)	
>2	1,325 (74.8)	447 (25.2)	
HLVA-IT critical scale score			
Responded “no” to 2^nd^ filter question	302 (64.1)	169 (35.9)	<0.001
1–2	37 (75.5)	12 (24.5)	
>2	1,324 (74.3)	457 (25.7)	
HLVA-IT communicative scale score			
Responded “no” to 2^nd^ filter question	302 (64.1)	169 (35.9)	<0.001
1–2	22 (81.5)	5 (18.5)	
>2	1,339 (74.3)	464 (25.7)	
Responder received a COVID-19 shot			
Yes	1,569 (73.1)	577 (26.9)	0.001
No	94 (60.65)	61 (39.35)	
Responder will vaccinate children<12y.o. with anti-COVID-19 vaccines (1,636)*			
Low prob.	250 (66.3)	127 (33.7)	0.001
Medium prob.	225 (76.5)	69 (23.5)	
High prob.	731 (75.75)	234 (24.25)	
Responder will vaccinate children>12y.o. with anti-COVID-19 vaccines (1,109)*			
Low probability	190 (63.8)	108 (36.2)	0.016
Medium probability	165 (73)	61 (27)	
High probability	424 (72.5)	161 (27.5)	

PACVs, Parent Attitudes About Childhood Vaccines Survey; HLVa-IT, Health Literacy about Vaccination of adults in ITalian; *Number of responders.

In contrast with the low prevalence of pediatric vaccinal hesitancy, and while most parents (93.3%) had already received at least one shot of the anti-COVID-19 vaccine, the 16.4% of them reported they were not likely to vaccinate their <12 yo children and 13% would not vaccinate their >12 yo children with anti-COVID-19 vaccines. Of note, almost the 40% of >12 yo children had already received at least one shot. In line with the vaccinal attitude, parents who had not received an anti-COVID-19 shot yet were more prone to turn to NIS (39.4% vs. 26.9%). The same were true for parents indicating a low probability of anti-COVID-19 vaccination both for <12 yo children (33.7% vs. 23.5 for medium probability and 24.3 for high probability) and for >12 yo children (36.2% vs. 27.0% for medium probability and 27.5% for high probability).

Parents involved in the study showed a good vaccine health literacy, especially in the communicative and critical domains. Overall, 1,886 parents answered the questions in the functional scale section, and 1,830 those in the communicative and critical sections. Respectively, 80.3, 77.4, and 78.3% of the parents who completed the HLVA-it domain questions scored >2 on the functional, critical, and communicative scale. Interestingly, parents who scored less than 2 in any domain or answered “no” to any of the filter questions were significantly more inclined to access NIS (pV < 0.001 in all domains).

### Predictors for the preferred source of information choice

The multivariable logistic regression model (Table 3) showed that males were more likely to use NIS (OR 1.42, 95% CI: 1.11–1.81), as well as pediatric vaccine hesitancy (OR 2.52, 95% CI: 1.79–3.57). Other predictors that showed a statistical higher likelihood to turn toward alternative sources of information were: living in Turin (OR 1.60, 95% CI: 1.29–2.00), being a single parent (OR 1.33, 95% CI: 1.02–1.74), a lower perceived quality of the healthcare system (OR 1.31, 95% CI: 1.06–1.62; OR 2.51, 95% CI: 1.59–3.96, for medium and low perceived quality, respectively), being atheist or agnostic (OR 1.32, 95% CI: 1.01–1.74), a higher child’s school grade (for middle school OR 1.69, 95% CI: 1.25–2.28; for high school OR 1.60, 95% CI: 1.21–2.12) and referring to require more information about vaccinations (OR 1.44, 95% CI: 1.20–1.75).

**Table 3:** Results of the multivariable logistic regression model to identify predictors of alternative information sources choice by parents about childhood vaccinations.

	OR	95% CI	*p*-Value
City of origin (Turin)	1.60	1.29–2.00	<0.001
Parental role (father)	1.48	1.15–1.90	0.002
Age (years, continuous)	1.01	0.99–1.03	0.359
Marital status (0 = single parent, 1 = married/cohabitant)	1.33	1.02–1.74	0.036
Profession (0 = employed, 1 = unemployed/housework)	1.26	0.91–1.74	0.168
Nationality (0 = Italian, 1 = other)	1.49	0.91–2.42	0.109
Religion (0 = believer)			
Non-believers	1.32	1.01–1.74	0.049
Would rather not answer/other	0.84	0.61–1.16	0.299
Pediatric vaccinal hesitancy (yes)	2.45	1.73–3.46	<0.001
Educational level (0 = compulsory schooling, 1 = degree/post-degree)	1.17	0.96–1.44	0.119
School grade (ref. elementary school)			
Kindergarten	1.27	0.87–1.85	0.219
Middle school	1.69	1.25–2.28	0.001
High school	1.60	1.21–2.12	0.001
Perceived healthcare system quality (ref high)			
Medium	1.31	1.06–1.62	0.014
Low	2.51	1.59–3.96	<0.001
Self-perceived need for further information (yes)	1.44	1.20–1.75	<0.001
HLVa-IT functional scale score (ref higher VL, score ≥ 2)			
“No” answer to filter question n.1	1.40	1.12–1.79	0.006
Low VL (score < 2)	1.58	1.04–2.38	0.030
HLVa-IT critical scale score (ref higher VL, score ≥ 2)			
“No” answer to filter question n.2	1.40	1.12–1.77	0.004
Low VL (score < 2)	0.73	0.37–1.47	0.381

HLVa-IT, Health Literacy about Vaccination of adults in ITalian; VL, vaccine literacy; filter question n. 1 = “Have you ever seen any informative material about vaccinations?”; filter question n. 2 = “Have ever been advised to get a vaccination shot?”.

With regard to the vaccinal health literacy scales, parents with a low functional vaccine health literacy and those who refer to have never read any vaccination material (1st filter question) were more likely to use NIS than the parents with an adequate literacy, while in the critical and communicative domains, only answering “no” to the second filter question (that investigates if they had ever been offered to receive a vaccine) is associated with higher likelihood to use NIS. No other predictors were associated with the use of NIS.

## Discussion

Vaccine hesitancy is a significant issue affecting modern society. With the evolution of mass media, the population’s approach to healthcare, and public health specifically, changed drastically. It has been estimated that two-thirds of the world’s population has access to the Internet. This opportunity, together with a growing distrust toward authorities, has facilitated the development and the dissemination of several alternative medicine theories and movements (24). Thus, understanding and discovering the predictors that lead to choosing alternative sources of information over official ones is very important to prevent and address the spread of fake news and growing distrust toward healthcare providers. Our study showed that the factors significantly associated with the choice of alternative sources of information were city of residence, sex, marital status, religion belief, school grade, level of vaccine hesitancy, low trust in the national healthcare system, and the need of further information on the topic.

Although the higher prevalence of NIS users in Turin than Rome is not reported in the current literature, it could be partly justified by the different intensity with which the COVID-19 pandemic hit northern Italy compared to the rest of the nation in the early phases (25) and its different management (26), possibly leading to a different general perception of institution as well as of institutional communication between the two cities. This novel finding, in our opinion, is worth further investigation, as part of a much-needed analysis of the long-term consequences of the COVID-19 pandemic on our communities (27).

Fathers seemed to be more associated with choosing alternative sources. Indeed, previous studies showed that men are usually less efficient than women in effectively managing their health and illness. Indeed, low health literacy levels fuel men’s reticence for health help-seeking, also enhanced by the absence of targeted health literacy promotion programs (28). In our study, fathers declared more often than mothers that they had never read any material about vaccinations (22.2% of fathers versus 17.1% of mothers, pV = 0.013) and overall achieved lower scores, especially in the critical and communicative domains (pV = 0.005 and pV = 0.056 respectively). Similarly, this dynamic might affect the role of fathers in their children’s healthcare management. When it comes to handling children’s health issues, mothers are usually more likely to take care of the matter and healthcare providers too tend to interact with mothers more than with fathers. Furthermore, fathers who received information about vaccinations from alternative sources (friends, mass media) are usually more discouraged toward them compared to the ones who got information from official healthcare sources (29). Addressing this issue before the baby’s birth positively affects immunization timeliness during infancy (30). Moreover, mothers and fathers do not always agree on the matter and when this happens, the child is less likely to receive the shots in time or at all (29). Henceforth, an essential component of antenatal care should address existing concerns regarding immunization and using healthcare visits during pregnancy to initiate the conversation between the future parents (29).

An association between a higher school grade and the preference of NIS was shown significant in the present analysis. This could be explained as, with the increasing age of the child and the passage from childhood to adolescence, parents start dealing with their children’s health in a more autonomous way, instead of involving the pediatrician, conversely with what happens during childhood. Furthermore, most of childhood vaccinations are usually administered during infancy and early childhood, partially explaining why a larger diffusion of IS is more common among parents of children attending primary school (31). This dynamic needs to be further investigated, considering the importance of acceptance of vaccines primarily administered during middle and high school, like HPV and anti-meningitis in children that had not been previously immunized (32–34). A previous study shows that parents who had consulted an official healthcare source to gather information about HPV had a higher HPV vaccine knowledge and acceptability (35). Moreover, this behavior in parents of high school students might reflect in the relatively high level of vaccine hesitancy showed by high degree students toward COVID-19 vaccines (36).

Further results from the logistic regression highlight the marital status as one of the predictors for the choice of NIS. Previous literature shows that coming from a single parent household is a predictor worldwide for incomplete or delayed childhood immunizations (37, 38). The preference for NIS could hence be considered one of the factors leading toward vaccine hesitancy in this particular population.

Vaccine hesitancy was strongly associated with choosing alternative sources of information about vaccinations. Through machine learning strategies, it was found that a higher hesitancy is associated with a more intense social media traffic, for most of the vaccinations (39). The relationship between vaccine hesitancy and independent research of information conducted mainly online has been a huge issue during the COVID pandemic, to the point that scientists coined the term “infodemic.” According to the WHO website, an infodemic is “too much information including false or misleading information in digital and physical environments during a disease outbreak. It causes confusion and risk-taking behaviors that can harm health. It also leads to mistrust in health authorities and undermines the public health response” (40). This issue becomes relevant for childhood vaccination because an infodemic can intensify people’s distrust toward healthcare, including previous health measures that the population had tolerated up to this moment. So, in what appears to be a vicious circle, skeptic patients tend not to trust official guidelines and look elsewhere to collect information, primarily online, increasing even more their level of vaccine hesitancy. A cross-sectional study in Lebanon in 2020 on conspiracy beliefs and vaccination intent for COVID-19 showed that trust in official information sources (WHO, MoPh, etc) increased vaccination intent against COVID-19 while trust in other sources (Whatsapp, Facebook) decreased it. Also following conspiracy theories such as believing in the “man-made virus” theory or the “business control” theory significantly reduced the likelihood of vaccination (41).

Another result worth of note involves the relationship between religious beliefs and the chosen sources of information. This issue has yet to be widely investigated, but it has become more relevant during the COVID-19 infodemic. From our results, parents who defined themselves atheists or agnostics were more likely to choose alternative sources of information than those who reported following traditional religions or would rather not answer the question. Previous research on the topic showed that spirituality with non-religious affiliation was associated with firmer beliefs in religious conspiracy theories and COVID-19 vaccine refusal (42). In contrast, members affiliated with religious organizations did not report religious conspiracy theories beliefs (42). As a reflection of Italian demographics, most of the responding parents who reported being believers were Christian, specifically Catholic. Recently, the effects of Pope Francis’ religious authority have been investigated by analyzing Twitter users’ attitudes toward COVID-19 vaccination after the Pope addressed the matter by inviting everyone to get an anti-COVID-19 shot in order to stem the virus diffusion (41). It resulted that, while the Pope’s appeal would not convince those with firm beliefs against vaccinations, the ones in favor of vaccination would be provided with additional arguments in the debate and those who are undecided and looking for information to decide on the area of their faith could be encouraged by their leader’s voice on the topic (43). It would be interesting to perform further investigations on the topic, assessing whether following religious norms can have as counterpart a spontaneous adherence to social and institutionalized norms too, as opposed to a significant refuse of blind acceptance of rules by non-believers, who hold a more skeptical vision and henceforth tend to explore their options from different sources before complying with a law. Furthermore, a low perceived quality of the healthcare system and a self-perceived need for further information tend to lead parents to seek information elsewhere, not being satisfied with the material they are provided with. A large amount of recent scientific literature is pointing to this direction, underlying how distrust toward authorities and healthcare providers is one of the most critical determinants of vaccine hesitancy. Positive attitudes, a higher perceived social support and a higher perceived behavioral control are associated with parent’s intention to vaccinate their children, suggesting the importance of trust-building interventions that promote pro-vaccine social norms (44). Moreover, parents usually want more information than they are getting and the lack of it leads to worry and regret about vaccination decisions. Poor communication and negative relationships with health workers could impact on vaccination decisions and parents generally found it difficult to know which vaccination information source to trust, feeling that most of the available information is biased and unbalanced (45).

Lastly, the association between low scores on the VL scales and the preference for NIS, which is in line with recent finding among healthcare professionals (46), may show that probably parents cannot properly assess the advice they are provided with, driving them to search for information elsewhere. This behavior could partially justify the relationship between vaccine literacy and vaccine acceptance (47), which is, on the contrary, lacking for the general health literacy (48). The answers to the two filter questions of the HLVa-IT scale, both significatively associated with the choice of NIS, investigate previous contacts of the parent with information provided by healthcare professionals or institutions (whether they had ever seen any vaccine related content or whether they had ever been advised to receive a vaccine). A negative answer to either of them highlights the necessity to further reach out to this population, not only by providing advice and information on the topic, but also by increasing awareness and offering tools for understanding and assessing autonomously the knowledge on the subject.

Our study has some limitations that must be considered. First of all, due to bureaucratic reasons, only the kindergartens of the city of Turin were included in the study. However, given the low number of parents included there is no modification in the results if they are removed from the analysis. Second, the HLVa-IT tool could not be included in the multivariable logistic regression model as a scale, because it contains two filter question that would have narrowed drastically the number of responders. Further analysis using a different instrument could provide a more complete insight on the influence that health literacy and vaccine health literacy play when choosing sources of information on medical topics. Third, the section regarding COVID-19 was constructed at the beginning of 2021 while the answers were collected from June to October 2021 when the epidemiological situation and the state of emergency rules had already changed multiple times. Specifically, the last part of the survey focused on parents’ perception toward anti COVID-19 vaccines for themselves and their children. The survey was conducted before the introduction of compulsory Green Pass for either work or leisure activities and before the official release of the license for anti-COVID-19 vaccinations in children <12 yo. Henceforth, the results might not reflect adequately the intercurrent situation in this ever-evolving field and were not included in the multiple logistic regression model. Lastly, given the voluntary nature of the survey, we could not exclude some degree of self-selection on the one hand of the parents more attentive to the issue of vaccination and with greater health and vaccination literacy, on the other of the parents more critical toward the benefits of vaccination and toward the more institutional information. This may have led to an overestimation of the association of some of the factors identified with the choice of information sources. However, we enrolled 28 schools of different grade ranging from kindergarten to high schools, covering from the downtown to the suburbs of Turin and Rome. Moreover, the response rate was consistent between Turin and Rome and between the school grades. Considering these factors, we believe that the responses collected give a fair representation of the population of parents involved in the study.

Our study also presents some important points of strength. First, it includes a large sample size, involving parents from two of the biggest Italian cities. Moreover, parental attitudes from all school grades were investigated, offering a panoramic view over the issue through a trustworthy channel provided within the institutional frame of school official registries. Lastly, we assessed the sources of information along with vaccine hesitancy among parents through the administration of reliable survey tools validated from the international literature.

## Conclusion

The last century has shown an unprecedented and exponential growth in science and technology. Communication among people has become much easier, with the introduction of radios, TVs and phones and the birth of the Internet, that has been developing nonstop during the past three decades. If, on one side, this improved people’s living conditions worldwide, on the other side, it opened a lot of new scenarios that need to be investigated and addressed as rapidly as this phenomenon is developing. Medicine and particularly public health need to be looked at from an anthropological point of view, given the growing complexity of modern society. For years, medical workers have been encouraging an informed kind of medicine, in which patients take initiative in their own healthcare management and decision making, with a broader boost of self-awareness. This process, though, led most people to autonomously search for the information they were looking for, not always being able to assess the quality of the content they would find. This study shows the importance of rebuilding a trust relationship between patients and health care providers, struggling between an overwhelmed and often fund and resource lacking healthcare system and the contemporary spread of fake news and disinformation, that find a fertile soil among the discouraged and skeptical portions of the population.

Moreover, public opinion is very cautious when it comes to children’s health and safety, to the point that, regarding vaccinations, the fear of side effects can sometimes swing opinions more than the undeniable role vaccines have in preventing severe infectious diseases that are becoming rarer thanks to the vaccination campaigns themselves. Henceforth, understanding parents, their fears, their opinions and what influences their choices regarding healthcare is of paramount importance to safeguard the present and future generations.

Shifting public attention from harms to benefits, in a rational evidence-based manner, should be one of the main goals of new information plans. This research showed that only 0.2% of parents got vaccination information from schools: thus, it could be smart to implement health promotion programs in schools, where children spend most of their time and to which usually parents are very careful to. This kind of interventions on one side could be very helpful and fruitful improving vaccine acceptance in both adults and children, on the other side, it could help develop the informed health managing process in a positive way, by educating children and favoring their health literacy from a very early stage. Finding new strategies to fight this battle can seem very challenging, and thus requires a conjoined effort among public health specialists, to prevent problems such as vaccine hesitancy and the spread of fake news that, if underestimated, could invalidate centuries of scientific and medical progress.

## Data availability statement

The raw data supporting the conclusions of this article will be made available by the authors, without undue reservation.

## Ethics statement

The studies involving humans were approved by Institutional Ethics Board of the Regione Lazio (Comitato Etico Lazio 1, protocol number 660, approved in date June 16th 2022). The studies were conducted in accordance with the local legislation and institutional requirements. The participants provided their written informed consent to participate in this study.

## Author contributions

RB, CV, and AM contributed to the conception and design of the study. RB, VC, FN, and MS performed data collection. RB and GM conducted the analyses. RB, GM, TS, and MS contributed to data curation. RB wrote the first draft of the manuscript. VC and GM wrote sections of the manuscript. RS, PV, CV, AM, and TS critically revised the manuscript. AM, GM, RS, PV, and CV contributed to supervision and funding acquisition. All authors contributed to manuscript revision and read and approved the submitted version.

## Conflict of interest

The authors declare that the research was conducted in the absence of any commercial or financial relationships that could be construed as a potential conflict of interest.

## Publisher’s note

All claims expressed in this article are solely those of the authors and do not necessarily represent those of their affiliated organizations, or those of the publisher, the editors and the reviewers. Any product that may be evaluated in this article, or claim that may be made by its manufacturer, is not guaranteed or endorsed by the publisher.
