# Supplementary material for: Who chooses alternative sources of information about childhood vaccinations? A cross-sectional study
Source: Front Public Health. 2023 Sep 13;11:1225761. doi: 10.3389/fpubh.2023.1225761 (PMC10525346; doi:10.3389/fpubh.2023.1225761)
Supplement: Supplementary file 1 [file Data_Sheet_1.docx]

Supplementary Material

Who chooses alternative sources of information about childhood vaccinations? A cross-sectional study

**Supplementary Material 1 – Questionnaire**

**Section A. Socio – demographic characteristics**

1. The present survey is compiled by:

Mother

Father

1. What year were you born in? (parent) _______
2. What’s your nationality?

Italian

Other: ______________

1. What’s your marital status?

Married

Separated

Divorced

Widow/er

Cohabitant

Single

1. What’s your occupation?

Full time employment

Part time employment

Self employed

Housewife

Looking for an employment

Unemployed

Other ________________

1. What’s your partner’s occupation?

Full time employment

Part time employment

Self employed

Housewife

Looking for an employment

Unemployed

Other _______________

1. What area does your job belong to?

Agriculture/fishing

Factory

Construction

Retail

Transport and logistics

Restaurant/hospitality

Information and communication

Finance and insurance services

Real estate

Healthcare associated profession/social care services

Art, sports and entertainment

Personal service activity

Other service activities (repairs, etc)

Family service activity (housekeeping, etc)

Other_________

1. What is your highest educational level:

None

Primary school

Middle school

High school

Bachelor Degree

Post-graduate

1. What is your partner’s highest educational level:

None

Primary school

Middle school

High school

Bachelor Degree

Post-graduate

1. When referring to politics, a lot of people use the terms “left” and “right”.

On a scale from 0 (strongly left wing) to 10 (strongly right wing), how would you describe your political tendencies? *(The question is not compulsory and the compiler can decide to skip it)*

(strongly left wing) 0 – 1 – 2 – 3 – 4 – 5 – 6 – 7 – 8 – 9 – 10 (strongly right wing)

1. Choose the box describing your religion:

Catholic

Orthodox

Jewish

Islamic

Jehovah’s witness

I would rather not answer the question

Other_____

1. On a scale from 0 (terrible) to 10 (excellent), how would you rate the National Healthcare System quality?

(terrible) 0 – 1 – 2 – 3 – 4 – 5 – 6 – 7 – 8 – 9 – 10 (excellent)

1. Do you have any other children besides the one this survey refers to?

No (*skip to section B*)

Yes, one

Yes, two

Yes, three

Yes, four

1. Other children age

|  | - 0 – 10 years | - 11 – 14 | - 15 – 18 | - Older than 18 | - Not applicable |
| --- | --- | --- | --- | --- | --- |
| - Second child |  |  |  |  |  |
| - Third child |  |  |  |  |  |
| - Fourth child |  |  |  |  |  |
| - Fifth child |  |  |  |  |  |

**Section B. Attitudes and behaviors**

*The questions in this section will evaluate your attitudes and your behavior regarding vaccines and refer to the son/daughter through whom you received this survey or to another son/daughter at least 18 months younger.*

**Questions from the PACV score.**

1. Please indicate month and year of birth of the child this survey refers to _______
2. Have you ever delayed having your child get a shot for reasons other than illness or allergy?

Yes

No (skip to question 20)

I don’t know (go to question 20)

1. For which of the following vaccines have you delayed your child’s shots?

| Anti- | Yes | no |
| --- | --- | --- |
| Diphtheria (hexavalent) |  |  |
| Tetanus (hexavalent) |  |  |
| Pertussis (hexavalent) |  |  |
| Haemophilus Influenzae B (hexavalent) |  |  |
| Hepatitis B (hexavalent) |  |  |
| Poliomyelitis (hexavalent) |  |  |
| Measles (MPR) |  |  |
| Mumps (MPR) |  |  |
| Rubella (MPR) |  |  |
| Chicken pox |  |  |
| Meningococcus B |  |  |
| Meningococcus C |  |  |
| Rotavirus |  |  |
| Pneumococcus |  |  |
| HPV |  |  |

1. For what reason? (*More than one answer allowed*)

|  | Yes | No |
| --- | --- | --- |
| Forgetfulness |  |  |
| Distance from vaccination centers |  |  |
| Vaccine not available at the vaccination center |  |  |
| Inconvenient vaccination center operating hours |  |  |
| Not recommended by the pediatrician |  |  |
| Other |  |  |

1. If you answered “Other” to the previous question, please specify the reason why you delayed the administration of the dose to your child _________________________
2. Have you ever decided not to have your child get a shot for reasons other than illness or allergy?

Yes

No (*skip to question 24*)

I don’t know (*skip to question 24*)

1. For which of the following vaccines have you refused your child’s shots?

| Anti - | Yes | no |
| --- | --- | --- |
| Diphtheria (hexavalent) |  |  |
| Tetanus (hexavalent) |  |  |
| Pertussis (hexavalent) |  |  |
| Haemophilus Influenzae B (hexavalent) |  |  |
| Hepatitis B (hexavalent) |  |  |
| Poliomyelitis (hexavalent) |  |  |
| Measles (MPR) |  |  |
| Mumps (MPR) |  |  |
| Rubella (MPR) |  |  |
| Chicken pox |  |  |
| Meningococcus B |  |  |
| Meningococcus C |  |  |
| Rotavirus |  |  |
| Pneumococcus |  |  |
| HPV |  |  |

1. For what reason? (*More than one answer allowed*)

|  | Yes | No |
| --- | --- | --- |
| Forgetfulness |  |  |
| Distance from vaccination centers |  |  |
| Vaccine not available at the vaccination center |  |  |
| Inconvenient vaccination center operating hours |  |  |
| Not recommended by the pediatrician |  |  |
| Other |  |  |

1. If you answered “Other” to the previous question, please specify the reason why you refused the administration of the dose to your child _________________________
2. How sure are you that following the recommended shot schedule is a good idea for your child?

*Please answer on a scale of 0 to 10, where 0 is Not at all sure and 10 is Completely sure.*

(not at all sure) 0 – 1 – 2 – 3 – 4 – 5 – 6 – 7 – 8 – 9 – 10 (completely sure)

1. Children get more shots than are good for them.

Strongly disagree

Disagree

Not sure

Agree

Strongly agree

1. I believe that many of the illnesses that shots prevent are severe.

Strongly disagree

Disagree

Not sure

Agree

Strongly agree

1. It is better for my child to develop immunity by getting sick than to get a shot.

Strongly disagree

Disagree

Not sure

Agree

Strongly agree

1. It is better for children to get fewer vaccines at the same time.

Strongly disagree

Disagree

Not sure

Agree

Strongly agree

1. How concerned are you that your child might have a serious side effect from a shot?

Very concerned

Somewhat concerned

Not sure

Not too concerned

Not at all concerned

1. How concerned are you that anyone of the childhood shots might not be safe?

Very concerned

Somewhat concerned

Not sure

Not too concerned

Not at all concerned

1. How concerned are you that a shot might not prevent the disease?

Very concerned

Somewhat concerned

Not sure

Not too concerned

Not at all concerned

1. If you had another infant today, would you want him/her to get all the recommended shots?

Yes

Don’t know

No

1. Overall, how hesitant about childhood shots would you consider yourself to be?

Very hesitant

Somewhat hesitant

Not too hesitant

Not at all hesitant

1. I trust the information I receive about shots.

Strongly disagree

Disagree

Not sure

Agree

Strongly agree

1. I am able to openly discuss my concerns about shots with my child’s doctor.

Strongly disagree

Disagree

Not sure

Agree

Strongly agree

1. All things considered, how much do you trust your child’s doctor?

*Please answer on a scale of 0 to 10, where 0 is Do not trust at all and 10 is Completely trust.*

(Do not trust at all) 0 – 1 – 2 – 3 – 4 – 5 – 6 – 7 – 8 – 9 – 10 (completely trust)

**Section C. Vaccine Literacy**

*The questions in this section of the survey are meant to evaluate the sources of information you access regarding vaccines.*

1. From which sources do you MAINLY acquire information regarding vaccines?

None

Family doctor

Pediatrician

Family, acquaintances, friends

School

TV, newspapers, reviews

Internet

Other ___________

1. Do you think you need further information regarding vaccines?

Yes

No

**Questions from the HLVa-IT** *English version -* ***H****ealth* ***L****iteracy about* ***V****accination in* ***a****dulthood in* ***It****alian (LR Biasio, C Giambi, G Fadda et al, Ann Ig 2020; 32(3): 205-222 - doi: 10.7416/ai.2020.2344)*

39. Have you ever read vaccine materials, such as leaflets or posters in doctor’s or public health offices, recommending vaccinations?

**□ NO □ YES – If yes, tick the boxes below, corresponding to your choice**

| **READING THE MATERIAL:**  (choose only one answer for each question) | **4-**  **Never** | **3-**  **Rarely** | **2-**  **Some times** | **1-**  **Often** |
| --- | --- | --- | --- | --- |
| 1. Did you find that the material as a whole  (texts and/or images) was difficult to read? | □ | □ | □ | □ |
| 2. Did he find words you didn’t know? | □ | □ | □ | □ |
| 3. Did you find that the texts were difficult to  understand? | □ | □ | □ | □ |
| 4. Did you need much time to understand them? | □ | □ | □ | □ |
| 5. Did you or would you need someone to help  you understand them? | □ | □ | □ | □ |

40. Have you ever thought or been advised to vaccinate yourself against one or more diseases?

**□ NO □ YES – If yes, tick the boxes below, corresponding to your choice**

| **WHEN SEARCHING INFORMATION:**  (choose only one answer for each question) | **1-**  **Never** | **2-**  **Rarely** | **3-**  **Some times** | **4-**  **Often** |
| --- | --- | --- | --- | --- |
| 6. Have you consulted more than one source of  information? | □ | □ | □ | □ |
| 7. Did you find the information you were  looking for? | □ | □ | □ | □ |
| 8. Did you understand the information found? | □ | □ | □ | □ |
| 9. Have you had the opportunity to use the  information? | □ | □ | □ | □ |
| 10. Did you discuss what you understood about vaccinations with your doctor or other  people? | □ | □ | □ | □ |
| 11. Did you consider whether the information  collected was about your condition? | □ | □ | □ | □ |
| 12. Have you considered the credibility of the  sources? | □ | □ | □ | □ |
| 13. Did you check whether the information was  correct? | □ | □ | □ | □ |
| 14. Did you find any useful information to make  a decision on whether or not to get vaccinated? | □ | □ | □ | □ |

**adapted from Ishikawa measure - Diabetes Care 2008, 31(5):874-879*

**Section D. COVID-19 Vaccine Hesitancy**

1. Have you received a COVID shot yet?

No, I haven’t been vaccinated (*skip to question 44*)

No, but I have a vaccination appointment scheduled (*skip to question 44*)

Yes, I received it because I work in healthcare (*skip to question 43*)

Yes, I received it because I work in school/in the army/ any other occupation prioritized category (*skip to question 43*)

Yes, I received it because I am older than 80/I live in a nursing home (*skip to question 43*)

Yes, I received it because I belong to an age bracket eligible for vaccination in my region (*skip to question 43*)

Yes, I received it because I belong to a fragile patient category (*skip to question 42*)

1. Please specify the fragile patient category you belong to __________
2. On a scale from 0 (poor) to 10 (excellent), how would you rate the information received from the healthcare providers during your vaccination appointment?

(poor) 0 – 1 – 2 – 3 – 4 – 5 – 6 – 7 – 8 – 9 – 10 (excellent)

1. Once you are eligible for it, on a scale from 0 to 100 how likely do you think you will be willing to receive the anti-COVID-19 shot?

0 % - 100 %

1. You selected a probability to get vaccinated lower than 100%, which one of the following statements is closer to the reason why today you are not completely sure about receiving a COVID-19 shot?

*(Be careful: you need to think about the reason why, despite having the possibility to receive the shot, you would be doubtful about it).*

Please select the MAIN, SECOND and THIRD REASON:

(*priority order*)

I don’t think the vaccines we have today are safe

I don’t think the vaccines we have today are effective

I don’t believe the specific vaccine designed for my category is safe

I don’t believe the specific vaccine designed for my category is effective

I don’t trust the sources of information that encourage vaccinations against COVID-19

I believe we have little trustworthy information about anti-COVID-19 vaccines

I know of serious adverse reactions that happened to family/acquaintances after receiving a Pfizer COVID shot

I know of serious adverse reactions that happened to family/acquaintances after receiving a Moderna COVID shot

I know of serious adverse reactions that happened to family/acquaintances after receiving an AstraZeneca COVID shot

I suffer from a condition that is not compatible with an anti-COVID-19 shot

I don’t believe in any vaccine at all and my opinion does not change when it comes to anti-COVID-19 shots

A person/source I trust encouraged me NOT to get a COVID shot

I don’t think I am at risk of contracting the infection

I don’t think the vaccines are effective against the new virus strains

I don’t think I am at risk of contracting a dangerous form of the infection

I already had a COVID-19 infection

I prefer to contract the infection organically

Other

1. You selected “Other” in the previous question. Please specify the reason why, despite having the possibility to receive a shot, you are not sure about vaccinating against COVID-19 _________________________________________
2. If you have children YOUNGER than 12, on a scale from 0 to 100, what is the likelihood you would vaccinate them against COVID-19, given the existence of a vaccine that is proven to be safe and effective?

- I don’t have any children younger than 12
- Scale 0% - 100%

1. You selected a probability lower than 100%, which one of the following statements is closer to the reason why today you are not completely sure about or you would rather not have your children receive a COVID-19 shot?

Please select the MAIN, SECOND and THIRD REASON:

*(priority order*)

I don’t think the vaccines we have today are safe

I don’t think the vaccines we have today are effective

I don’t trust the sources of information that encourage vaccinations against COVID-19

I know of serious adverse reactions that happened to family/acquaintances after receiving a COVID shot

COVID-19 infection is not that severe in children

I don’t believe in any vaccine at all and my opinion does not change when it comes to anti-COVID-19 shots

I would rather have my children receive only the vaccines required to attend school

Children receive too many vaccinations already

Vaccines increase the risk of developing conditions like autism, multiple sclerosis or diabetes

Vaccines increase the risk of developing allergies in children

My child has already contracted a COVID-19 infection

Other

1. You selected “Other” in the previous question. Please specify the reason why today you would not vaccinate or are not sure about vaccinating your children against COVID-19 ______________________________________________
2. If you have children with an age BETWEEN 12 and 17, on a scale from 0 to 100, what is the likelihood you would vaccinate them against COVID-19, now that these vaccines have been authorized for this category?

I don’t have any children in that age range

Scale 0% - 100%

My child has received the shot already

1. You selected a probability lower than 100%, which one of the following statements is closer to the reason why today you are not completely sure about or you would rather not have your children receive a COVID-19 shot?

Please select the MAIN, SECOND and THIRD REASON:

*(priority order*)

I don’t think the vaccines we have today are safe

I don’t think the vaccines we have today are effective

I don’t trust the sources of information that encourage vaccinations against COVID-19

I know of serious adverse reactions that happened to family/acquaintances after receiving a COVID shot

COVID-19 infection is not that severe in children

I don’t believe in any vaccine at all and my opinion does not change when it comes to anti-COVID-19 shots

I would rather have my children receive only the vaccines required to attend school

Children receive too many vaccinations already

Vaccines increase the risk of developing conditions like autism, multiple sclerosis or diabetes

Vaccines increase the risk of developing allergies in children

My child has already contracted a COVID-19 infection

Other

1. You selected “Other” in the previous question. Please specify the reason why today you would not vaccinate or are not sure about vaccinating your children against COVID-19 ______________________________________________
2. If, in order to keep its efficacy over time against the different viral strains, the administration of anti-COVID-19 shots became recommended yearly (similarly to flu shots), what is the likelihood you would get vaccinated?

0% - 100%

1. Has the global COVID-19 pandemic influenced your beliefs regarding vaccinations in general (excluding COVID-19 shots)?

No, my opinion regarding vaccinations has not changed

Yes, I am MORE inclined to vaccinate myself and/or my children

No, I am LESS inclined to vaccinate myself and/or my children
